# Patterns of Comorbidity and In-Hospital Mortality in Older Patients With COVID-19 Infection

**DOI:** 10.3389/fmed.2021.726837

**Published:** 2021-09-17

**Authors:** Mona Mahmoud, Luca Carmisciano, Luca Tagliafico, Mariya Muzyka, Gianmarco Rosa, Alessio Signori, Matteo Bassetti, Alessio Nencioni, Fiammetta Monacelli, The GECOVID Study Group

**Affiliations:** ^1^Geriatrics Clinic, Department of Internal Medicine and Medical Specialties (DIMI), University of Genoa, Genoa, Italy; ^2^Istituto di Ricovero e Cura a Carattere Scientifico Policlinico San Martino Hospital, Genoa, Italy; ^3^DISSAL, Department of Health Science, University of Genoa, Genoa, Italy; ^4^Cardiology Clinic, Department of Internal Medicine and Medical Specialties, University of Genoa, Genoa, Italy; ^5^Infectious Diseases Clinic, Department of Health Sciences, University of Genoa and Policlinico San Martino Hospital, Genoa, Italy

**Keywords:** COVID-19 infection, cluster of comorbidity, older adults, mortality, prognosis

## Abstract

**Introduction:** Older adults are more susceptible to severe COVID-19, with increased all-cause mortality. This has been attributed to their multimorbidity and disability. However, it remains to be established which clinical features of older adults are associated with severe COVID-19 and mortality. This information would aid in an accurate prognosis and appropriate care planning. Here, we aimed to identify the chronic clinical conditions and the comorbidity clusters associated with in-hospital mortality in a cohort of older COVID-19 patients who were admitted to the IRCCS Policlinico San Martino Hospital, Genoa, Italy, between January and April 2020.

**Methods:** This was a retrospective cohort study including 219 consecutive patients aged 70 years or older and is part of the GECOVID-19 study group. During the study period, upon hospital admission, demographic information (age, sex) and underlying chronic medical conditions (multimorbidity) were recorded from the medical records at the time of COVID-19 diagnosis before any antiviral or antibiotic treatment was administered. The primary outcome measure was in-hospital mortality.

**Results:** The vast majority of the patients (90%) were >80 years; the mean patient age was 83 ± 6.2 years, and 57.5% were men. Hypertension and cardiovascular disease, along with dementia, cerebrovascular diseases, and vascular diseases were the most prevalent clinical conditions. Multimorbidity was assessed with the Cumulative Illness Rating Scale. The risk of in-hospital mortality due to COVID-19 was higher for males, for older patients, and for patients with dementia or cerebral-vascular disease. We clustered patients into three groups based on their comorbidity pattern: the *Metabolic-renal-cancer* cluster, the *Neurocognitive* cluster and the *Unspecified* cluster. The *Neurocognitive* and *Metabolic-renal-cancer* clusters had a higher mortality compared with the *Unspecified* cluster, independent of age and sex.

**Conclusion:** We defined patterns of comorbidity that accurately identified older adults who are at higher risk of death from COVID-19. These associations were independent of chronological age, and we suggest that the identification of comorbidity clusters that have a common pathophysiology may aid in the early assessment of COVID-19 patients with frailty to promote timely interventions that, in turn, may result in a significantly improved prognosis.

## Introduction

At present, the global COVID-19 pandemic has reached a total of 174,809,365 confirmed cases worldwide^1^. Despite the widespread incidence of COVID-19 infection across different age groups, older adults have a higher susceptibility to severe COVID-19 with higher all-cause mortality and thus represent a major burden for health care systems (1). In particular, nursing home residents have been the most vulnerable group in terms of developing severe COVID-19 and dying from this disease. This has been attributed to the prevalence of chronic diseases (multimorbidity) and disability in this age group (2). Impaired immune function as a result of immunosenescence, which is a hallmark of frail older adults, is considered to be another key factor predisposing older adults who live in nursing homes to the most severe clinical outcomes (3).

Early studies observed that age, cancer, chronic obstructive pulmonary disease, chronic kidney failure or an immunocompromised state were associated with in-hospital mortality in older individuals (4, 5).

Diabetes mellitus, hypertension, cardiovascular diseases, and obesity were also identified as chronic clinical conditions associated with a higher risk of all-cause mortality in older adults with COVID-19 (4, 5).

Consistent with these data, older people with multimorbidity (i.e., the co-occurrence of three or more chronic clinical conditions) have been proposed to be at the highest risk for developing complications and for succumbing to COVID-19.

Older adults typically show broad heterogeneity in terms of their health status, and chronological age is considered to be a poor descriptor of their biological health status. In line with that, even multimorbidity *per se* is frequently inaccurate in defining an older person's biological status. Multimorbidity is heterogeneous among older individuals and may not accurately reflect clinical vulnerability or frailty, especially in very old patients. Indeed, multimorbidity may be considered normal in very old individuals, reaching a prevalence of 90% (6). Although multimorbidity has been consistently associated with unfavorable clinical outcomes in geriatric patients, its specificity is highly debated. Accordingly, the identification of specific comorbidity clusters that occur beyond chance is a meaningful way to reduce older adults' clinical complexity and holds promise for reflecting the biological aging of older individuals. Such an approach aims to identify diseases that co-occur in the same person, as they share a common pathophysiological background, that exceed a level expected by chance.

Whether specific comorbidity clusters and severe COVID-19 are associated in older individuals and whether these clusters are predictive of worse clinical outcomes, including mortality, remains largely unexplored. An understanding of this might help to define COVID-19 pathophysiology in older adults and allow for more appropriate interventions in a timely manner.

Here, we aimed to identify comorbidity clusters associated with in-hospital mortality in a cohort of older COVID-19 patients who were admitted to hospital during the first pandemic wave.

## Methods

### Subjects

This was a retrospective cohort study that included 219 consecutive patients aged 70 years and older who were admitted to the infectious disease clinical ward of the IRCCS Policlinico San Martino Hospital, Genoa, Italy, between January and April 2020. This study is part of the GECOVID-19 study group, which aims to improve the care of COVID-19 patients and conducts research according to a pre-established clinical form (6).

During the study period, older adults with COVID-19 were admitted to the hospital if they presented PA = 2 <60 mmHG at rest in ambient air or if the exacerbation of their underlying diseases or severe symptoms were considered unmanageable at home.

Patients were included in the study if they were at least 70 years old, were diagnosed with COVID [first clinical sample recorded as positive for severe acute respiratory syndrome coronavirus 2 (SARS-CoV-2) RNA], and had not previously received antiviral or antibiotic treatment for the infection.

Patients who signed a do-not-resuscitate document and who declined invasive treatment were excluded.

During the study period (first pandemic wave), upon hospital admission, demographic information (age, sex), and underlying chronic medical conditions were recorded from the medical records at the time of COVID diagnosis (i.e., at the time of the first clinical sample recorded as positive for SARS-CoV-2) and before any antiviral or antibiotic treatment was administered. A confirmed case of COVID-19 was defined by a positive result of an RT-PCR assay from a respiratory sample. Multimorbidity was assessed with the Cumulative Illness Rating Scale (CIRS) (7) (illustrated in Appendix 1). CIRS-C estimated the burden of multimorbidity and CIRS- S estimated the severity of chronic clinical conditions that are part of multimorbidity. The primary outcome measure was in-hospital mortality. The study protocol was approved by the Ethics Committee of Liguria region (N.CER 114/2020-ID), and the need for written informed consent was waived because of the retrospective nature of the study.

### Statistical Analysis

The mean, median, standard deviation (SD) and interquartile range (IQR) were used to summarize continuous variables; counts and percentages were used to summarize categorical variables. The *t*-test and Mann–Whitney test were used to detect differences in continuous variables between two groups, and the chi-squared test was used for categorical variables.

Multivariate logistic regression was used to estimate the association of clusters with mortality while adjusting for age, sex, and CIRS-C and CIRS-S. The odds ratio (OR), the 95% confidence interval (95% CI) and the *p*-values are reported.

Bernoulli Latent Block Model (LBM) was used for cluster analysis.

The risk ratio (RR) was used to describe the likelihood of being classified by LBM into each cluster compared to the others, with each cluster having specific anamnestic characteristics.

A two-sided α < 0.05 was considered statistically significant. R software version 4.0.2 was used for all statistical analyses (8). *P* < 0.05 were considered significant.

### Clustering Algorithm

Co-clustering is a technique for sorting heterogeneous data into homogeneous blocks and is used for clustering cases and variables simultaneously (rows and columns of a matrix). Co-clustering has the advantage of providing a direct interpretation of each cluster (9).

Clustering maximizes the distance between groups of COVID-19 patients and groups of their anamnestic baseline characteristics while increasing the similarity within each group; no information collected after COVID-19 infection was used to define the clusters.

The clustering model used (LBM) is a fully unsupervised probabilistic model for co-clustering that works with a matrix of homogeneous data—a set of binary anamnestic variables (the occurrence of each comorbidity). We used the pseudo-likelihood parameter as the criteria to select the optimal number of latent classes and clusters to detect.

## Results

The mean patient age was 83 ± 6.2 years. The vast majority of the patients (90%) were >80 years. Men accounted for 57.5% of the study subjects. The clinical characteristics of the patients are presented in Table 1.

**Table 1:** Patient clinical characteristics.

	**Overall, *N* = 219**
**Males**, ***N*****(%)**	126 (57.5)
**Age (years), Mean (** ***SD*** **)**	83 (6.2)
**Older age group (>80 y)**, ***N*****(%)**	90 (41.1)
**Multimorbidity**, ***N*****(%)**	
Hypertension	136 (62.1)
Cardiologic diseases	83 (37.9)
Dementia or cerebral-vascular disease	66 (30.1)
Vascular disease	59 (26.9)
Diabetes	40 (18.3)
Renal disease	40 (18.3)
Any type of cancer	37 (16.9)
Chronic obstructive pulmonary disease	24 (11.0)
Hepatic disease	10 (4.6)
HIV	3 (1.4)
CIRS-C,^*^Median [IQR]	4 [2, 5]
CIRS-S, ^**^Median [IQR]	1.7 [1.4, 2.0]
LoS ^***^(days), Median [IQR]	13 [6, 26]
In-hospital mortality, *N* (%)	132 (60.3)

* *CIRS-C multimorbidity*.

** *CIRS-S severity of multimorbidity*.

*** *LoS, length of hospital stay*.

Hypertension and cardiovascular disease, along with dementia, cerebrovascular diseases, and vascular diseases, were the most prevalent clinical conditions. The mean CIRS score was 4 ± 2.5, indicating moderate multimorbidity.

The mean in-hospital stay was 13 days, and in-hospital mortality was 60.3%.

The risk of in-hospital mortality due to COVID-19 was higher for men (OR = 2.1; 95%CI 1.2, 4.0; *p* = 0.017), for older patients (>85 years vs. younger OR = 2.0; 95%CI 1.1, 3.9; *p* = 0.026) and for patients with dementia or cerebral-vascular disease (OR = 2.3; 95%CI 1.2, 4.7; *p* = 0.017), adjusted for CIRS-C and CIRS-S (Table 2).

**Table 2:** Multivariable binomial logistic results modeling the risk of death.

	**Model 1**		**Model 2**	
**Term**	**OR (95%CI)**	***p***	**OR (95%CI)**	***p***
**Cluster**				
Unspecified	ref	–	ref	–
Metabolic-renal-cancer	2.9 (1.1, 8.2)	0.035^*^	2.9 (1.1, 8.1)	0.040^*^
Neurocognitive	2.8 (1.4, 5.8)	0.005^*^	2.8 (1.4, 5.7)	0.006^*^
**Sex (male vs. female)**	2.1 (1.2, 3.8)	0.015^*^	2.1 (1.2, 3.8)	0.016^*^
**Age group (above 85 years vs. younger)**	2.3 (1.2, 4.2)	0.008^*^	2.3 (1.3, 4.2)	0.008^*^
**CIRS-C**	0.9 (0.8, 1.1)	0.425	–	–
**CIRS-S**	–	–	0.7 (0.3, 1.8)	0.472

* *Means statistically significant*.

**Figure 1:**
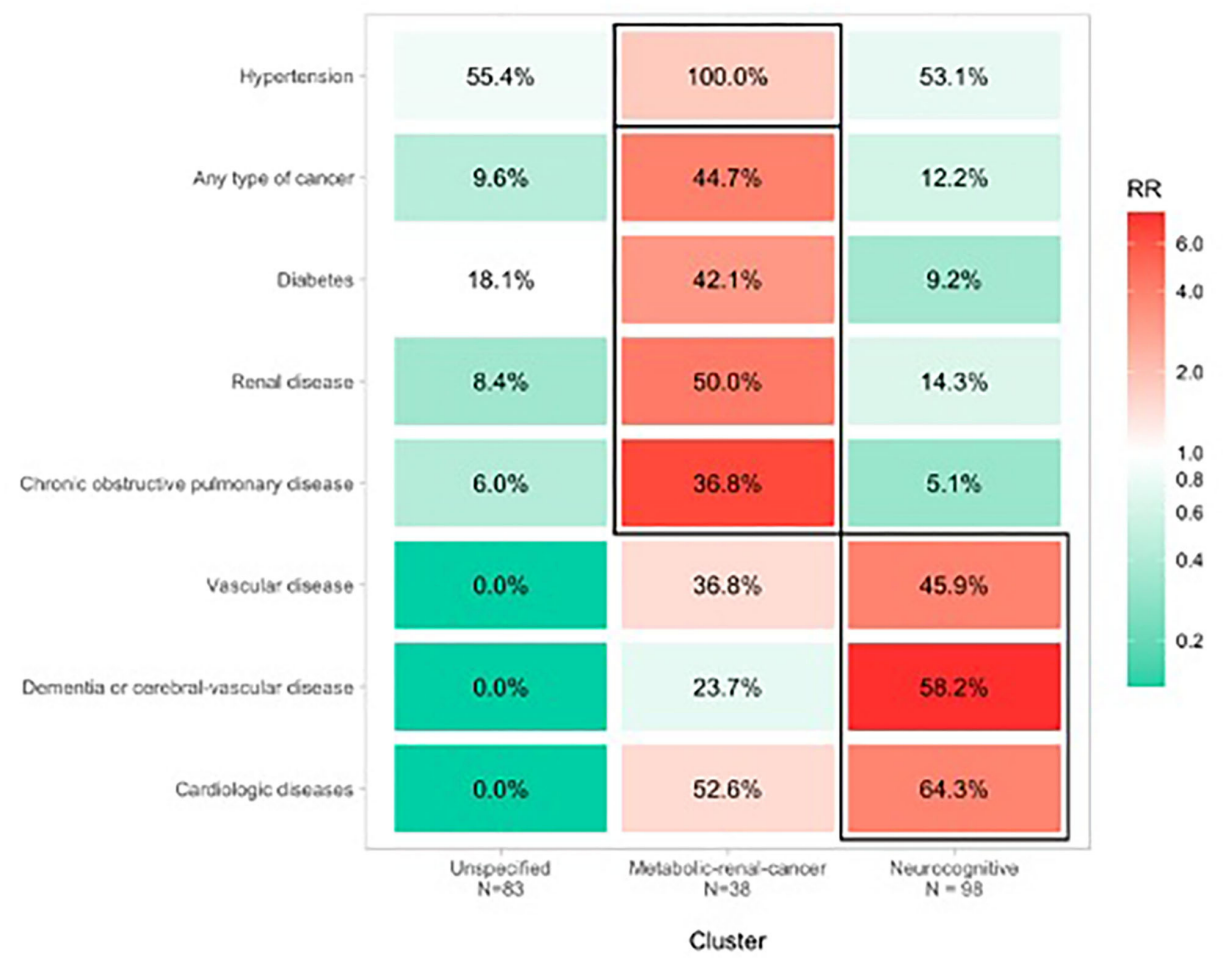
Clusters of comorbidity associated with COVID-19 mortality in hospitalized older patients.

We clustered patients into three groups based on their comorbidity pattern (Figure 1). Compared with the other clusters, the *Metabolic-renal-cancer* cluster had higher proportions of patients with hypertension (all patients; RR = 1.82; 95%CI 1.59, 2.09), diabetes (42.1% prevalence; RR = 3.18; 95%CI 1.87, 5.38), kidney disease (50% prevalence, RR = 4.31; 95%CI 2.58, 7.20), previous active cancer (44.7% prevalence; RR = 4.05; 95%CI 2.35, 6.97), and chronic obstructive pulmonary disease (36.8% prevalence; RR = 6.67; 95%CI 3.21, 13.87).

Compared with the other clusters, the *Neurocognitive* cluster had higher proportions of patients with heart disease (64.3% prevalence; RR = 3.89; 95% CI 2.54, 5.96), dementia (58.2% prevalence; RR = 7.82; 95% CI 4.08, 14.99), and cerebral-vascular disease or vascular diseases (45.9% prevalence; RR = 3.97; 95% CI 2.32, 6.79).

The *Unspecified* cluster included different chronic clinical conditions with prevalences ranging between 0 and 9% (except for hypertension, which was present in 55.4% of patients) and RRs ranging from 0.01 to 0.98.

In both the *Neurocognitive* and *Metabolic-renal-cancer* clusters, higher mortality was observed compared with the *Unspecified* cluster (chi-squared = 11.7, *p* = 0.003). Patients in the *Neurocognitive* and *Metabolic-renal-cancer* clusters had an ~2.4-fold increased risk of dying compared with those in the *Unspecified* cluster, independent of age and sex (Figure 1).

Clusters were not associated with sex (chi-squared = 2.66, *p* = 0.264) or age (chi-squared = 1.36, *p* = 0.506).

In addition, the CIRS interaction in the cluster analysis was adjusted based on low multimorbidity (CIRS <4) and moderate to high multimorbidity (CIRS >4) (Appendix 2 in the Supplementary Material) and additionally the CIRS-S interaction with cluster analysis was also performed on the basis of CIRS severity (CIRS-S <2; CIRS -S >2). Namely, a CIRS-C interaction for comorbidity cluster was found in the *Neurocognitive* cluster, although a general trend was observed in all clusters based on the levels of multimorbidity (CIRS-C) and CIRS-S severity.

## Discussion

In this study, we performed a methodologically robust analysis of comorbidity clusters associated with COVID-19-related mortality in a sample of hospitalized older patients.

The data indicate that COVID-19-related mortality was higher in patients with dementia and cerebrovascular disease, and these conditions retained an independent association with all-cause in-hospital mortality. In addition, patients at higher risk of dying from COVID-19 were clustered into two main groups: the *Neurocognitive* cluster, which was characterized by dementia, cerebral-vascular disease or vascular diseases and cardiologic diseases, and the *Metabolic-renal-cancer* cluster, which was characterized by hypertension, diabetes, renal diseases, previous or active cancer, and chronic obstructive respiratory disease. Notably, age and sex were not associated with clustering, supporting the notion that chronological age poorly reflects biological aging.

Recently, in a large Italian case series, Vetrano et al. (10) showed that specific comorbidities, including cardiovascular diseases, metabolic conditions and dementia, co-occurred in older patients who died from COVID-19. These clusters of comorbidities may be a clinical indication of worse COVID-19 prognosis. In particular, heart failure, metabolic conditions, stroke, and dementia were associated with greater odds of hospitalization, intensive care need, and mortality.

Several studies have found that the levels of inflammatory markers are increased in both COVID-19 patients and in frail individuals, indicating that the inflammatory response may be involved in the worsened clinical course of COVID-19 in older and frail patients (11, 12).

COVID-19 has emerged as a potent inducer of systemic, uncontrolled inflammation, which can worsen pre-existing conditions (e.g., heart failure, kidney failure, COPD, dementia) and, ultimately, frail patients often succumb to this severe infection owing to multisystem damage and failure to thrive (12–14).

In line with previous findings (10), the *Neurocognitive* cluster and the *Metabolic-renal-cancer* cluster had stronger associations with COVID-19 in-hospital mortality.

In the *Neurocognitive* cluster, dementia and stroke emerged as a particular disease clustering with the highest mortality. Dementia often develops as the result of stroke, and both conditions share common cardiovascular risk factors (10). In addition, both dementia and stroke are known risk factors for advanced clinical frailty in very old individuals, reflecting the massive burden of diseases related to severe COVID-19 complications and mortality (15). Similarly, the presence of cardiovascular diseases may be responsible for an impaired hemodynamic response to COVID-19 infection with the development of acute organ decompensation, poorer cardiovascular fitness, and higher rate of hospitalization and unfavorable clinical outcomes, including mortality (10–16).

In the *Metabolic-renal-cancer* cluster, there was a high likelihood of the co-occurrence of diabetes, COPD and kidney disease. Arguably, the presence of immunodeficiencies as a result of chronic inflammation has been extensively observed in diabetes and metabolic syndrome, promoting impaired coagulation, endothelial dysfunction, insulin resistance and oxidative stress and potentially contributing to a rapidly evolving COVID-19 disease course (17).

Similarly, an underlying impaired respiratory fitness, as in the case of COPD, may account for the rapid deterioration of COVID-19 patients. However, among smokers, there is a high degree of inter-individual variability in the development of lethal complications from COVID-19 (18).

Moreover, an impaired renal hemodynamic response as a result of the presence of hypertension, atherosclerotic diseases, cardiovascular, and/or metabolic conditions may account for more severe COVID-19 and a rapidly evolving disease course (19). Indeed, individuals with underlying kidney disease may be particularly vulnerable to COVID-19 illness marked by multisystem organ failure, thrombosis and higher inflammatory response, conferring a unique risk for rapid evolving cases beyond that related to multimorbidity burden (19).

Another interesting finding is the higher likelihood to underscore the disease combination of cancer.

However, although cancer patients displayed higher clinical vulnerability, the effects of various confounding factors, such as the presence of underlying comorbidities, including a suppressed immune system and a hypercoagulable state are difficult to separate from the effects of having cancer. In addition, presenting symptoms of COVID-19 infection and common symptoms of both cancer and toxicity from anticancer therapy, making the diagnosis even more difficult (20).

At present, there is no clinical understanding of the pathogenesis of very poor COVID-19-related outcomes and frailty in older adults, nor are there recognized interventions or measurements for this a highly vulnerable population (13).

However, our findings are in keeping with the large data analysis conducted by the Italian Superior Institute of Health (ISS) (10). That analysis found that cardiovascular disease, metabolic conditions, COPD, stroke and dementia were the most frequent diseases associated with COVID-19-related mortality in older patients.

Labenz et al. (21, 22) recently found that the clinical frailty scale (CFS), independent of age and multimorbidity, could predict worse COVID-19 progression and higher mortality in older adults. Thus, routine CSF testing upon hospital admission in persons aged 65 years and more may identify at-risk patients, and it might be a suitable risk marker for increased mortality. Frail patients (CSF 6-9) of all ages had a higher in-hospital mortality than fit patients. However, there was no significant difference in mortality between mildly frail patients (CSF 4-5) and fit patients admitted to intensive care units. Therefore, the identification of a combined tool including CSF stratification for frailty and a specific set of comorbidities that share a common pathogenesis may help to refine an appropriate assessment to identify highly vulnerable patients who require more medical resources and high levels of care in an early and timely manner.

A strength of this study is the use of real-world data from older patients hospitalized for COVID-19. In addition, although preliminary, these findings improve our ability to identify clusters of comorbidities that may contribute to a higher risk of dying from SARS-CoV-2 infection, irrespective of chronological age. These specific clusters of diseases are considered to contribute to more severe COVID-19 and the rapid evolution of the disease course, ultimately leading to death.

This study was limited by the retrospective nature of the analysis and the by the single time point assessment of a small sample size. Moreover, the data were collected in the first pandemic wave during which there was poor knowledge of the disease and related treatments. This may affect the generalizability of the findings. The use of in-hospital data also reduces the generalizability of the findings to other care settings. To better define COVID-19 pathophysiology, prevention and treatment strategies, additional longitudinal studies with larger populations are needed to further evaluate the interactions between clusters of patients, diseases, and unfavorable clinical outcomes associated with COVID-19.

## Conclusions and Future Challenges

A thorough clinical understanding to provide the appropriate assessment and early identification of older adults with frailty who are prone to worsened COVID complications with unfavorable clinical outcomes is still needed (13, 21). At present, there is an insufficient clinical understanding of the exact pathogenesis of frailty in older adults with COVID-19 (13).

COVID-19 strongly interacts with frailty in very old individuals in a vicious cycle. The identification of comorbidity clusters that have a common pathophysiology may aid in the early assessment of COVID-19 patients with frailty to promote timely interventions that, in turn, may result in a significantly improved prognosis.

Defining patients' comorbidity patterns, beyond chronological age, may reflect that impaired adaptative immune responses and increased inflammaging are important factors in the early recognition of the biological state of patients who are highly prone to rapid clinical deterioration or mortality. Identifying these clusters may improve the interventions used in this vulnerable population by improving clinician awareness.

The findings of this study have advanced our understanding of this issue and, although preliminary, may have important clinical implications. Indeed, the future definition of an algorithm that includes comorbidity clustering, frailty status, and markers of inflammation and immunosenescence may help to disentangle the detrimental interaction between frailty, very old age, and poor COVID-19 prognosis and help to establish multicomponent interventions for frailty and for the modulation of the therapeutic aggressiveness of COVID-19 treatments.

Investigating the associations between COVID-19 mortality, biological aging, and comorbidity patterns holds promise for delineating the true risk of COVID-19 mortality in older adults.

## Data Availability Statement

The raw data supporting the conclusions of this article will be made available by the authors, without undue reservation.

## Ethics Statement

The studies involving human participants were reviewed and approved by Ethics Committee of Liguria region (N.CER 114/2020-ID). Written informed consent for participation was not required for this study in accordance with the national legislation and the institutional requirements.

## Author Contributions

MMa and LC: conceptualization and validation. LT: validation. AS: methodology, software, and formal statistical analysis. MB: data curation. MMu: investigation, data analysis, and interpretation. AN and GR: writing, review, and editing. FM: writing, review, editing, and supervision. All authors contributed to the article and approved the submitted version.

## Funding

This work was supported in part by the Associazione Italiana per la Ricerca sul Cancro (AIRC, #17736 and #22098, to AN), by the Italian Ministry of Health (PE-2016-02363073; to FM) and by the University of Genoa.

## Conflict of Interest

The authors declare that the research was conducted in the absence of any commercial or financial relationships that could be construed as a potential conflict of interest.

## Publisher's Note

All claims expressed in this article are solely those of the authors and do not necessarily represent those of their affiliated organizations, or those of the publisher, the editors and the reviewers. Any product that may be evaluated in this article, or claim that may be made by its manufacturer, is not guaranteed or endorsed by the publisher.

## Footnotes

^1^ https://www.worldometers.info/coronavirus/
