# Supplementary material for: Patterns of Comorbidity and In-Hospital Mortality in Older Patients With COVID-19 Infection
Source: Front Med (Lausanne). 2021 Sep 17;8:726837. doi: 10.3389/fmed.2021.726837 (PMC8486012; doi:10.3389/fmed.2021.726837)
Supplement: Supplementary file 1 [file Table_1.DOCX]

Appendix 1. Age and sex adjusted risk of in hospital mortality for clusters

|  | OR (95%CI) | p value |
| --- | --- | --- |
| Age group (≥85 years vs <85 years) | 2.21 (1.22, 4.09) | 0.010 |
| Sex (male vs female) | 2.15 (1.20, 3.90) | 0.010 |
| Cluster |  |  |
| Unspecified | Reference | - |
| Metabolic-renal-cancer | 2.35 (1.04, 5.53) | 0.045 |
| Neurocognitive | 2.43 (1.31, 4.59) | 0.005 |
