# Supplementary material for: Patterns of Comorbidity and In-Hospital Mortality in Older Patients With COVID-19 Infection
Source: Front Med (Lausanne). 2021 Sep 17;8:726837. doi: 10.3389/fmed.2021.726837 (PMC8486012; doi:10.3389/fmed.2021.726837)
Supplement: Supplementary file 2 [file Table_2.docx]

**Collaborators**

- **GECOVID study group**:

[Anna Alessandrini](https://pubmed.ncbi.nlm.nih.gov/?sort=date&term=Alessandrini+A) [^9^](https://pubmed.ncbi.nlm.nih.gov/32810610/#affiliation-9), [Marco Camera](https://pubmed.ncbi.nlm.nih.gov/?sort=date&term=Camera+M) [^9^](https://pubmed.ncbi.nlm.nih.gov/32810610/#affiliation-9), [Emanuele Delfino](https://pubmed.ncbi.nlm.nih.gov/?sort=date&term=Delfino+E) [^9^](https://pubmed.ncbi.nlm.nih.gov/32810610/#affiliation-9), [Andrea De Maria](https://pubmed.ncbi.nlm.nih.gov/?sort=date&term=De+Maria+A) [^9^](https://pubmed.ncbi.nlm.nih.gov/32810610/#affiliation-9), [Chiara Dentone](https://pubmed.ncbi.nlm.nih.gov/?sort=date&term=Dentone+C) [^9^](https://pubmed.ncbi.nlm.nih.gov/32810610/#affiliation-9), [Antonio Di Biagio](https://pubmed.ncbi.nlm.nih.gov/?sort=date&term=Di+Biagio+A) [^9^](https://pubmed.ncbi.nlm.nih.gov/32810610/#affiliation-9), [Ferdinando Dodi](https://pubmed.ncbi.nlm.nih.gov/?sort=date&term=Dodi+F) [^9^](https://pubmed.ncbi.nlm.nih.gov/32810610/#affiliation-9), [Antonio Ferrazin](https://pubmed.ncbi.nlm.nih.gov/?sort=date&term=Ferrazin+A) [^9^](https://pubmed.ncbi.nlm.nih.gov/32810610/#affiliation-9), [Giovanni Mazzarello](https://pubmed.ncbi.nlm.nih.gov/?sort=date&term=Mazzarello+G) [^9^](https://pubmed.ncbi.nlm.nih.gov/32810610/#affiliation-9), [Malgorzata Mikulska](https://pubmed.ncbi.nlm.nih.gov/?sort=date&term=Mikulska+M) [^9^](https://pubmed.ncbi.nlm.nih.gov/32810610/#affiliation-9), [Laura Nicolini](https://pubmed.ncbi.nlm.nih.gov/?sort=date&term=Nicolini+L) [^9^](https://pubmed.ncbi.nlm.nih.gov/32810610/#affiliation-9), [Federica Toscanini](https://pubmed.ncbi.nlm.nih.gov/?sort=date&term=Toscanini+F) [^9^](https://pubmed.ncbi.nlm.nih.gov/32810610/#affiliation-9), [Daniele R Giacobbe](https://pubmed.ncbi.nlm.nih.gov/?sort=date&term=Giacobbe+DR) [^9^](https://pubmed.ncbi.nlm.nih.gov/32810610/#affiliation-9), [Antonio Vena](https://pubmed.ncbi.nlm.nih.gov/?sort=date&term=Vena+A) [^9^](https://pubmed.ncbi.nlm.nih.gov/32810610/#affiliation-9), [Lucia Taramasso](https://pubmed.ncbi.nlm.nih.gov/?sort=date&term=Taramasso+L) [^9^](https://pubmed.ncbi.nlm.nih.gov/32810610/#affiliation-9), [Elisa Balletto](https://pubmed.ncbi.nlm.nih.gov/?sort=date&term=Balletto+E) [^9^](https://pubmed.ncbi.nlm.nih.gov/32810610/#affiliation-9), [Federica Portunato](https://pubmed.ncbi.nlm.nih.gov/?sort=date&term=Portunato+F) [^9^](https://pubmed.ncbi.nlm.nih.gov/32810610/#affiliation-9), [Eva Schenone](https://pubmed.ncbi.nlm.nih.gov/?sort=date&term=Schenone+E) [^9^](https://pubmed.ncbi.nlm.nih.gov/32810610/#affiliation-9), [Nirmala Rosseti](https://pubmed.ncbi.nlm.nih.gov/?sort=date&term=Rosseti+N) [^9^](https://pubmed.ncbi.nlm.nih.gov/32810610/#affiliation-9), [Federico Baldi](https://pubmed.ncbi.nlm.nih.gov/?sort=date&term=Baldi+F) [^9^](https://pubmed.ncbi.nlm.nih.gov/32810610/#affiliation-9), [Marco Berruti](https://pubmed.ncbi.nlm.nih.gov/?sort=date&term=Berruti+M) [^9^](https://pubmed.ncbi.nlm.nih.gov/32810610/#affiliation-9), [Federica Briano](https://pubmed.ncbi.nlm.nih.gov/?sort=date&term=Briano+F) [^9^](https://pubmed.ncbi.nlm.nih.gov/32810610/#affiliation-9), [Silvia Dettori](https://pubmed.ncbi.nlm.nih.gov/?sort=date&term=Dettori+S) [^9^](https://pubmed.ncbi.nlm.nih.gov/32810610/#affiliation-9), [Laura Labate](https://pubmed.ncbi.nlm.nih.gov/?sort=date&term=Labate+L) [^9^](https://pubmed.ncbi.nlm.nih.gov/32810610/#affiliation-9), [Laura Magnasco](https://pubmed.ncbi.nlm.nih.gov/?sort=date&term=Magnasco+L) [^9^](https://pubmed.ncbi.nlm.nih.gov/32810610/#affiliation-9), [Michele Mirabella](https://pubmed.ncbi.nlm.nih.gov/?sort=date&term=Mirabella+M) [^9^](https://pubmed.ncbi.nlm.nih.gov/32810610/#affiliation-9), [Rachele Pincino](https://pubmed.ncbi.nlm.nih.gov/?sort=date&term=Pincino+R) [^9^](https://pubmed.ncbi.nlm.nih.gov/32810610/#affiliation-9), [Chiara Russo](https://pubmed.ncbi.nlm.nih.gov/?sort=date&term=Russo+C) [^9^](https://pubmed.ncbi.nlm.nih.gov/32810610/#affiliation-9), [Giovanni Sarteschi](https://pubmed.ncbi.nlm.nih.gov/?sort=date&term=Sarteschi+G) [^9^](https://pubmed.ncbi.nlm.nih.gov/32810610/#affiliation-9), [Chiara Sepulcri](https://pubmed.ncbi.nlm.nih.gov/?sort=date&term=Sepulcri+C) [^9^](https://pubmed.ncbi.nlm.nih.gov/32810610/#affiliation-9), [Stefania Tutino](https://pubmed.ncbi.nlm.nih.gov/?sort=date&term=Tutino+S) [^9^](https://pubmed.ncbi.nlm.nih.gov/32810610/#affiliation-9), [Roberto Pontremoli](https://pubmed.ncbi.nlm.nih.gov/?sort=date&term=Pontremoli+R) [^10^](https://pubmed.ncbi.nlm.nih.gov/32810610/#affiliation-10), [Valentina Beccati](https://pubmed.ncbi.nlm.nih.gov/?sort=date&term=Beccati+V) [^10^](https://pubmed.ncbi.nlm.nih.gov/32810610/#affiliation-10), [Salvatore Casciaro](https://pubmed.ncbi.nlm.nih.gov/?sort=date&term=Casciaro+S) [^10^](https://pubmed.ncbi.nlm.nih.gov/32810610/#affiliation-10), [Massimo Casu](https://pubmed.ncbi.nlm.nih.gov/?sort=date&term=Casu+M) [^10^](https://pubmed.ncbi.nlm.nih.gov/32810610/#affiliation-10), [Francesco Gavaudan](https://pubmed.ncbi.nlm.nih.gov/?sort=date&term=Gavaudan+F) [^10^](https://pubmed.ncbi.nlm.nih.gov/32810610/#affiliation-10), [Maria Ghinatti](https://pubmed.ncbi.nlm.nih.gov/?sort=date&term=Ghinatti+M) [^10^](https://pubmed.ncbi.nlm.nih.gov/32810610/#affiliation-10), [Elisa Gualco](https://pubmed.ncbi.nlm.nih.gov/?sort=date&term=Gualco+E) [^10^](https://pubmed.ncbi.nlm.nih.gov/32810610/#affiliation-10), [Giovanna Leoncini](https://pubmed.ncbi.nlm.nih.gov/?sort=date&term=Leoncini+G) [^10^](https://pubmed.ncbi.nlm.nih.gov/32810610/#affiliation-10), [Paola Pitto](https://pubmed.ncbi.nlm.nih.gov/?sort=date&term=Pitto+P) [^10^](https://pubmed.ncbi.nlm.nih.gov/32810610/#affiliation-10), [Kassem Salam](https://pubmed.ncbi.nlm.nih.gov/?sort=date&term=Salam+K) [^10^](https://pubmed.ncbi.nlm.nih.gov/32810610/#affiliation-10), [Angelo Gratarola](https://pubmed.ncbi.nlm.nih.gov/?sort=date&term=Gratarola+A) [^11^](https://pubmed.ncbi.nlm.nih.gov/32810610/#affiliation-11), [Mattia Bixio](https://pubmed.ncbi.nlm.nih.gov/?sort=date&term=Bixio+M) [^11^](https://pubmed.ncbi.nlm.nih.gov/32810610/#affiliation-11), [Annalisa Amelia](https://pubmed.ncbi.nlm.nih.gov/?sort=date&term=Amelia+A) [^11^](https://pubmed.ncbi.nlm.nih.gov/32810610/#affiliation-11), [Andrea Balestra](https://pubmed.ncbi.nlm.nih.gov/?sort=date&term=Balestra+A) [^11^](https://pubmed.ncbi.nlm.nih.gov/32810610/#affiliation-11), [Paola Ballarino](https://pubmed.ncbi.nlm.nih.gov/?sort=date&term=Ballarino+P) [^11^](https://pubmed.ncbi.nlm.nih.gov/32810610/#affiliation-11), [Nicholas Bardi](https://pubmed.ncbi.nlm.nih.gov/?sort=date&term=Bardi+N) [^11^](https://pubmed.ncbi.nlm.nih.gov/32810610/#affiliation-11), [Roberto Boccafogli](https://pubmed.ncbi.nlm.nih.gov/?sort=date&term=Boccafogli+R) [^11^](https://pubmed.ncbi.nlm.nih.gov/32810610/#affiliation-11), [Francesca Caserza](https://pubmed.ncbi.nlm.nih.gov/?sort=date&term=Caserza+F) [^11^](https://pubmed.ncbi.nlm.nih.gov/32810610/#affiliation-11), [Elisa Calzolari](https://pubmed.ncbi.nlm.nih.gov/?sort=date&term=Calzolari+E) [^11^](https://pubmed.ncbi.nlm.nih.gov/32810610/#affiliation-11), [Marta Castelli](https://pubmed.ncbi.nlm.nih.gov/?sort=date&term=Castelli+M) [^11^](https://pubmed.ncbi.nlm.nih.gov/32810610/#affiliation-11), [Elisabetta Cenni](https://pubmed.ncbi.nlm.nih.gov/?sort=date&term=Cenni+E) [^11^](https://pubmed.ncbi.nlm.nih.gov/32810610/#affiliation-11), [Paolo Cortese](https://pubmed.ncbi.nlm.nih.gov/?sort=date&term=Cortese+P) [^11^](https://pubmed.ncbi.nlm.nih.gov/32810610/#affiliation-11), [Giuseppe Cuttone](https://pubmed.ncbi.nlm.nih.gov/?sort=date&term=Cuttone+G) [^11^](https://pubmed.ncbi.nlm.nih.gov/32810610/#affiliation-11), [Sara Feltrin](https://pubmed.ncbi.nlm.nih.gov/?sort=date&term=Feltrin+S) [^11^](https://pubmed.ncbi.nlm.nih.gov/32810610/#affiliation-11), [Stefano Giovinazzo](https://pubmed.ncbi.nlm.nih.gov/?sort=date&term=Giovinazzo+S) [^11^](https://pubmed.ncbi.nlm.nih.gov/32810610/#affiliation-11), [Patrizia Giuntini](https://pubmed.ncbi.nlm.nih.gov/?sort=date&term=Giuntini+P) [^11^](https://pubmed.ncbi.nlm.nih.gov/32810610/#affiliation-11), [Letizia Natale](https://pubmed.ncbi.nlm.nih.gov/?sort=date&term=Natale+L) [^11^](https://pubmed.ncbi.nlm.nih.gov/32810610/#affiliation-11), [Davide Orsi](https://pubmed.ncbi.nlm.nih.gov/?sort=date&term=Orsi+D) [^11^](https://pubmed.ncbi.nlm.nih.gov/32810610/#affiliation-11), [Matteo Pastorino](https://pubmed.ncbi.nlm.nih.gov/?sort=date&term=Pastorino+M) [^11^](https://pubmed.ncbi.nlm.nih.gov/32810610/#affiliation-11), [Tommaso Perazzo](https://pubmed.ncbi.nlm.nih.gov/?sort=date&term=Perazzo+T) [^11^](https://pubmed.ncbi.nlm.nih.gov/32810610/#affiliation-11), [Fabio Pescetelli](https://pubmed.ncbi.nlm.nih.gov/?sort=date&term=Pescetelli+F) [^11^](https://pubmed.ncbi.nlm.nih.gov/32810610/#affiliation-11), [Federico Schenone](https://pubmed.ncbi.nlm.nih.gov/?sort=date&term=Schenone+F) [^11^](https://pubmed.ncbi.nlm.nih.gov/32810610/#affiliation-11), [Maria G Serra](https://pubmed.ncbi.nlm.nih.gov/?sort=date&term=Serra+MG) [^11^](https://pubmed.ncbi.nlm.nih.gov/32810610/#affiliation-11), [Marco Sottano](https://pubmed.ncbi.nlm.nih.gov/?sort=date&term=Sottano+M) [^11^](https://pubmed.ncbi.nlm.nih.gov/32810610/#affiliation-11), [Roberto Tallone](https://pubmed.ncbi.nlm.nih.gov/?sort=date&term=Tallone+R) [^12^](https://pubmed.ncbi.nlm.nih.gov/32810610/#affiliation-12), [Massimo Amelotti](https://pubmed.ncbi.nlm.nih.gov/?sort=date&term=Amelotti+M) [^12^](https://pubmed.ncbi.nlm.nih.gov/32810610/#affiliation-12), [Marie J Majabò](https://pubmed.ncbi.nlm.nih.gov/?sort=date&term=Majab%C3%B2+MJ) [^12^](https://pubmed.ncbi.nlm.nih.gov/32810610/#affiliation-12), [Massimo Merlini](https://pubmed.ncbi.nlm.nih.gov/?sort=date&term=Merlini+M) [^12^](https://pubmed.ncbi.nlm.nih.gov/32810610/#affiliation-12), [Federica Perazzo](https://pubmed.ncbi.nlm.nih.gov/?sort=date&term=Perazzo+F) [^12^](https://pubmed.ncbi.nlm.nih.gov/32810610/#affiliation-12), [Nidal Ahamd](https://pubmed.ncbi.nlm.nih.gov/?sort=date&term=Ahamd+N) [^13^](https://pubmed.ncbi.nlm.nih.gov/32810610/#affiliation-13), [Paolo Barbera](https://pubmed.ncbi.nlm.nih.gov/?sort=date&term=Barbera+P) [^13^](https://pubmed.ncbi.nlm.nih.gov/32810610/#affiliation-13), [Marta Bovio](https://pubmed.ncbi.nlm.nih.gov/?sort=date&term=Bovio+M) [^13^](https://pubmed.ncbi.nlm.nih.gov/32810610/#affiliation-13), [Paola Campodonico](https://pubmed.ncbi.nlm.nih.gov/?sort=date&term=Campodonico+P) [^13^](https://pubmed.ncbi.nlm.nih.gov/32810610/#affiliation-13), [Andrea Collidà](https://pubmed.ncbi.nlm.nih.gov/?sort=date&term=Collid%C3%A0+A) [^13^](https://pubmed.ncbi.nlm.nih.gov/32810610/#affiliation-13), [Ombretta Cutuli](https://pubmed.ncbi.nlm.nih.gov/?sort=date&term=Cutuli+O) [^13^](https://pubmed.ncbi.nlm.nih.gov/32810610/#affiliation-13), [Agnese Lomeo](https://pubmed.ncbi.nlm.nih.gov/?sort=date&term=Lomeo+A) [^13^](https://pubmed.ncbi.nlm.nih.gov/32810610/#affiliation-13), [Francesca Fezza](https://pubmed.ncbi.nlm.nih.gov/?sort=date&term=Fezza+F) [^13^](https://pubmed.ncbi.nlm.nih.gov/32810610/#affiliation-13), [Nicola Gentilucci](https://pubmed.ncbi.nlm.nih.gov/?sort=date&term=Gentilucci+N) [^13^](https://pubmed.ncbi.nlm.nih.gov/32810610/#affiliation-13), [Nadia Hussein](https://pubmed.ncbi.nlm.nih.gov/?sort=date&term=Hussein+N) [^13^](https://pubmed.ncbi.nlm.nih.gov/32810610/#affiliation-13), [Emanuele Malvezzi](https://pubmed.ncbi.nlm.nih.gov/?sort=date&term=Malvezzi+E) [^13^](https://pubmed.ncbi.nlm.nih.gov/32810610/#affiliation-13), [Laura Massobrio](https://pubmed.ncbi.nlm.nih.gov/?sort=date&term=Massobrio+L) [^13^](https://pubmed.ncbi.nlm.nih.gov/32810610/#affiliation-13), [Giula Motta](https://pubmed.ncbi.nlm.nih.gov/?sort=date&term=Motta+G) [^13^](https://pubmed.ncbi.nlm.nih.gov/32810610/#affiliation-13), [Laura Pastorino](https://pubmed.ncbi.nlm.nih.gov/?sort=date&term=Pastorino+L) [^13^](https://pubmed.ncbi.nlm.nih.gov/32810610/#affiliation-13), [Nicoletta Pollicardo](https://pubmed.ncbi.nlm.nih.gov/?sort=date&term=Pollicardo+N) [^13^](https://pubmed.ncbi.nlm.nih.gov/32810610/#affiliation-13), [Stefano Sartini](https://pubmed.ncbi.nlm.nih.gov/?sort=date&term=Sartini+S) [^13^](https://pubmed.ncbi.nlm.nih.gov/32810610/#affiliation-13), [Paola Vacca](https://pubmed.ncbi.nlm.nih.gov/?sort=date&term=Vacca+P) [^13^](https://pubmed.ncbi.nlm.nih.gov/32810610/#affiliation-13), [Valentina Virga](https://pubmed.ncbi.nlm.nih.gov/?sort=date&term=Virga+V) [^13^](https://pubmed.ncbi.nlm.nih.gov/32810610/#affiliation-13), [Italo Porto](https://pubmed.ncbi.nlm.nih.gov/?sort=date&term=Porto+I) [^14^](https://pubmed.ncbi.nlm.nih.gov/32810610/#affiliation-14), [Giampaolo Bezante](https://pubmed.ncbi.nlm.nih.gov/?sort=date&term=Bezante+G) [^14^](https://pubmed.ncbi.nlm.nih.gov/32810610/#affiliation-14), [Roberta Della Bona](https://pubmed.ncbi.nlm.nih.gov/?sort=date&term=Della+Bona+R) [^14^](https://pubmed.ncbi.nlm.nih.gov/32810610/#affiliation-14), [Giovanni La Malfa](https://pubmed.ncbi.nlm.nih.gov/?sort=date&term=La+Malfa+G) [^14^](https://pubmed.ncbi.nlm.nih.gov/32810610/#affiliation-14), [Alberto Valbusa](https://pubmed.ncbi.nlm.nih.gov/?sort=date&term=Valbusa+A) [^14^](https://pubmed.ncbi.nlm.nih.gov/32810610/#affiliation-14), [Vered G Ad](https://pubmed.ncbi.nlm.nih.gov/?sort=date&term=Ad+VG) [^14^](https://pubmed.ncbi.nlm.nih.gov/32810610/#affiliation-14), [Emanuela Barisione](https://pubmed.ncbi.nlm.nih.gov/?sort=date&term=Barisione+E) [^15^](https://pubmed.ncbi.nlm.nih.gov/32810610/#affiliation-15), [Michele Bellotti](https://pubmed.ncbi.nlm.nih.gov/?sort=date&term=Bellotti+M) [^15^](https://pubmed.ncbi.nlm.nih.gov/32810610/#affiliation-15), [Aloe' Teresita](https://pubmed.ncbi.nlm.nih.gov/?sort=date&term=Teresita+A) [^15^](https://pubmed.ncbi.nlm.nih.gov/32810610/#affiliation-15), [Alessandro Blanco](https://pubmed.ncbi.nlm.nih.gov/?sort=date&term=Blanco+A) [^15^](https://pubmed.ncbi.nlm.nih.gov/32810610/#affiliation-15), [Marco Grosso](https://pubmed.ncbi.nlm.nih.gov/?sort=date&term=Grosso+M) [^15^](https://pubmed.ncbi.nlm.nih.gov/32810610/#affiliation-15), [Maria Grazia Piroddi](https://pubmed.ncbi.nlm.nih.gov/?sort=date&term=Piroddi+MG) [^15^](https://pubmed.ncbi.nlm.nih.gov/32810610/#affiliation-15), [Paolo Moscatelli](https://pubmed.ncbi.nlm.nih.gov/?sort=date&term=Moscatelli+P) [^16^](https://pubmed.ncbi.nlm.nih.gov/32810610/#affiliation-16), [Paola Ballarino](https://pubmed.ncbi.nlm.nih.gov/?sort=date&term=Ballarino+P) [^16^](https://pubmed.ncbi.nlm.nih.gov/32810610/#affiliation-16), [Matteo Caiti](https://pubmed.ncbi.nlm.nih.gov/?sort=date&term=Caiti+M) [^16^](https://pubmed.ncbi.nlm.nih.gov/32810610/#affiliation-16), [Elisabetta Cenni](https://pubmed.ncbi.nlm.nih.gov/?sort=date&term=Cenni+E) [^16^](https://pubmed.ncbi.nlm.nih.gov/32810610/#affiliation-16), [Patrizia Giuntini](https://pubmed.ncbi.nlm.nih.gov/?sort=date&term=Giuntini+P) [^16^](https://pubmed.ncbi.nlm.nih.gov/32810610/#affiliation-16), [Ottavia Magnani](https://pubmed.ncbi.nlm.nih.gov/?sort=date&term=Magnani+O) [^16^](https://pubmed.ncbi.nlm.nih.gov/32810610/#affiliation-16), [Samir Sukkar](https://pubmed.ncbi.nlm.nih.gov/?sort=date&term=Sukkar+S) [^17^](https://pubmed.ncbi.nlm.nih.gov/32810610/#affiliation-17), [Ludovica Cogorno](https://pubmed.ncbi.nlm.nih.gov/?sort=date&term=Cogorno+L) [^17^](https://pubmed.ncbi.nlm.nih.gov/32810610/#affiliation-17), [Raffaella Gradaschi](https://pubmed.ncbi.nlm.nih.gov/?sort=date&term=Gradaschi+R) [^17^](https://pubmed.ncbi.nlm.nih.gov/32810610/#affiliation-17), [Erica Guiddo](https://pubmed.ncbi.nlm.nih.gov/?sort=date&term=Guiddo+E) [^17^](https://pubmed.ncbi.nlm.nih.gov/32810610/#affiliation-17), [Eleonora Martino](https://pubmed.ncbi.nlm.nih.gov/?sort=date&term=Martino+E) [^17^](https://pubmed.ncbi.nlm.nih.gov/32810610/#affiliation-17), [Livia Pisciotta](https://pubmed.ncbi.nlm.nih.gov/?sort=date&term=Pisciotta+L) [^17^](https://pubmed.ncbi.nlm.nih.gov/32810610/#affiliation-17), [Bruno Cavagliere](https://pubmed.ncbi.nlm.nih.gov/?sort=date&term=Cavagliere+B) [^18^](https://pubmed.ncbi.nlm.nih.gov/32810610/#affiliation-18), [Rossi Cristina](https://pubmed.ncbi.nlm.nih.gov/?sort=date&term=Cristina+R) [^18^](https://pubmed.ncbi.nlm.nih.gov/32810610/#affiliation-18), [Farina Francesca](https://pubmed.ncbi.nlm.nih.gov/?sort=date&term=Francesca+F) [^18^](https://pubmed.ncbi.nlm.nih.gov/32810610/#affiliation-18), [Giacomo Garibotto](https://pubmed.ncbi.nlm.nih.gov/?sort=date&term=Garibotto+G) [^19^](https://pubmed.ncbi.nlm.nih.gov/32810610/#affiliation-19), [Pasquale Esposito](https://pubmed.ncbi.nlm.nih.gov/?sort=date&term=Esposito+P) [^19^](https://pubmed.ncbi.nlm.nih.gov/32810610/#affiliation-19), [Carmen Bellezza](https://pubmed.ncbi.nlm.nih.gov/?sort=date&term=Bellezza+C) [^20^](https://pubmed.ncbi.nlm.nih.gov/32810610/#affiliation-20), [Emirjona Harusha](https://pubmed.ncbi.nlm.nih.gov/?sort=date&term=Harusha+E) [^20^](https://pubmed.ncbi.nlm.nih.gov/32810610/#affiliation-20), [Francesca Rossi](https://pubmed.ncbi.nlm.nih.gov/?sort=date&term=Rossi+F) [^20^](https://pubmed.ncbi.nlm.nih.gov/32810610/#affiliation-20), [Eleonora Arboscello](https://pubmed.ncbi.nlm.nih.gov/?sort=date&term=Arboscello+E) [^20^](https://pubmed.ncbi.nlm.nih.gov/32810610/#affiliation-20), [Laura Arzani](https://pubmed.ncbi.nlm.nih.gov/?sort=date&term=Arzani+L) [^20^](https://pubmed.ncbi.nlm.nih.gov/32810610/#affiliation-20), [Laura De Mattei](https://pubmed.ncbi.nlm.nih.gov/?sort=date&term=De+Mattei+L) [^20^](https://pubmed.ncbi.nlm.nih.gov/32810610/#affiliation-20), [Marzia Spadaro](https://pubmed.ncbi.nlm.nih.gov/?sort=date&term=Spadaro+M) [^20^](https://pubmed.ncbi.nlm.nih.gov/32810610/#affiliation-20), [Giovanni Passalacqua](https://pubmed.ncbi.nlm.nih.gov/?sort=date&term=Passalacqua+G) [^21^](https://pubmed.ncbi.nlm.nih.gov/32810610/#affiliation-21), [Diego Bagnasco](https://pubmed.ncbi.nlm.nih.gov/?sort=date&term=Bagnasco+D) [^21^](https://pubmed.ncbi.nlm.nih.gov/32810610/#affiliation-21), [Fulvio Braido](https://pubmed.ncbi.nlm.nih.gov/?sort=date&term=Braido+F) [^21^](https://pubmed.ncbi.nlm.nih.gov/32810610/#affiliation-21), [Annamaria Riccio](https://pubmed.ncbi.nlm.nih.gov/?sort=date&term=Riccio+A) [^21^](https://pubmed.ncbi.nlm.nih.gov/32810610/#affiliation-21), [Elena Tagliabue](https://pubmed.ncbi.nlm.nih.gov/?sort=date&term=Tagliabue+E) [^21^](https://pubmed.ncbi.nlm.nih.gov/32810610/#affiliation-21), [Claudio Gustavino](https://pubmed.ncbi.nlm.nih.gov/?sort=date&term=Gustavino+C) [^22^](https://pubmed.ncbi.nlm.nih.gov/32810610/#affiliation-22), [Antonella Ferraiolo](https://pubmed.ncbi.nlm.nih.gov/?sort=date&term=Ferraiolo+A) [^22^](https://pubmed.ncbi.nlm.nih.gov/32810610/#affiliation-22), [Fiammetta Monacelli](https://pubmed.ncbi.nlm.nih.gov/?sort=date&term=Monacelli+F) [^23^](https://pubmed.ncbi.nlm.nih.gov/32810610/#affiliation-23), [Mona Mahmoud](https://pubmed.ncbi.nlm.nih.gov/?sort=date&term=Mahmoud+M) [^23^](https://pubmed.ncbi.nlm.nih.gov/32810610/#affiliation-23), [Luca Tagliafico](https://pubmed.ncbi.nlm.nih.gov/?sort=date&term=Tagliafico+L) [^23^](https://pubmed.ncbi.nlm.nih.gov/32810610/#affiliation-23), [Armando Napolitano](https://pubmed.ncbi.nlm.nih.gov/?sort=date&term=Napolitano+A) [^23^](https://pubmed.ncbi.nlm.nih.gov/32810610/#affiliation-23), [Maria Fiorio](https://pubmed.ncbi.nlm.nih.gov/?sort=date&term=Fiorio+M) [^23^](https://pubmed.ncbi.nlm.nih.gov/32810610/#affiliation-23), [Monica Pizzonia](https://pubmed.ncbi.nlm.nih.gov/?sort=date&term=Pizzonia+M) [^23^](https://pubmed.ncbi.nlm.nih.gov/32810610/#affiliation-23), [Chiara Giannotti](https://pubmed.ncbi.nlm.nih.gov/?sort=date&term=Giannotti+C) [^23^](https://pubmed.ncbi.nlm.nih.gov/32810610/#affiliation-23), [Alessio Nencioni](https://pubmed.ncbi.nlm.nih.gov/?sort=date&term=Nencioni+A) [^23^](https://pubmed.ncbi.nlm.nih.gov/32810610/#affiliation-23), [Salvatore Giuffrida](https://pubmed.ncbi.nlm.nih.gov/?sort=date&term=Giuffrida+S) [^24^](https://pubmed.ncbi.nlm.nih.gov/32810610/#affiliation-24), [Nicola Rosso](https://pubmed.ncbi.nlm.nih.gov/?sort=date&term=Rosso+N) [^24^](https://pubmed.ncbi.nlm.nih.gov/32810610/#affiliation-24), [Alessandra Morando](https://pubmed.ncbi.nlm.nih.gov/?sort=date&term=Morando+A) [^25^](https://pubmed.ncbi.nlm.nih.gov/32810610/#affiliation-25), [Riccardo Papalia](https://pubmed.ncbi.nlm.nih.gov/?sort=date&term=Papalia+R) [^25^](https://pubmed.ncbi.nlm.nih.gov/32810610/#affiliation-25), [Donata Passerini](https://pubmed.ncbi.nlm.nih.gov/?sort=date&term=Passerini+D) [^25^](https://pubmed.ncbi.nlm.nih.gov/32810610/#affiliation-25), [Gabriella Tiberio](https://pubmed.ncbi.nlm.nih.gov/?sort=date&term=Tiberio+G) [^25^](https://pubmed.ncbi.nlm.nih.gov/32810610/#affiliation-25), [Giovanni Orengo](https://pubmed.ncbi.nlm.nih.gov/?sort=date&term=Orengo+G) [^26^](https://pubmed.ncbi.nlm.nih.gov/32810610/#affiliation-26), [Alberto Battaglini](https://pubmed.ncbi.nlm.nih.gov/?sort=date&term=Battaglini+A) [^26^](https://pubmed.ncbi.nlm.nih.gov/32810610/#affiliation-26), [Silvano Ruffoni](https://pubmed.ncbi.nlm.nih.gov/?sort=date&term=Ruffoni+S), [Sergio Caglieris](https://pubmed.ncbi.nlm.nih.gov/?sort=date&term=Caglieris+S)
