# Supplementary material for: Patterns of Comorbidity and In-Hospital Mortality in Older Patients With COVID-19 Infection
Source: Front Med (Lausanne). 2021 Sep 17;8:726837. doi: 10.3389/fmed.2021.726837 (PMC8486012; doi:10.3389/fmed.2021.726837)
Supplement: Supplementary file 3 [file Table_3.docx]

Appendix 2: Sensitivity analysis, CIRS-C and CIRS_S interactions

| Term | CIRS-C | | | CIRS-S | | |
| --- | --- | --- | --- | --- | --- | --- |
|  | **CIRS-C ≤4,**  **N = 97** | **CIRS-C >4,**  **N = 122** | **p*** | **CIRS-S ≤2,**  **N = 157** | **CIRS-S >2,**  **N = 62** | **p*** |
|  | **OR (95%CI)** | **OR (95%CI)** |  | **OR (95%CI)** | **OR (95%CI)** |  |
| Cluster |  |  |  |  |  |  |
| Unspecified | ref | ref | - | ref | ref | - |
| Metabolic-renal-cancer | 2.9 (0.3, 62.1) | 1.5 (0.5, 4.9) | 0.572 | 2.4 (0.7, 9.8) | 1.2 (0.0, 14.7) | 0.525 |
| Neurocognitive | 6.2 (2.2, 20.2) | 1.3 (0.4, 3.6) | 0.042* | 3.1 (1.5, 6.5) | 1.0 (0.0, 11.5) | 0.309 |
| Sex  (Male vs Female) | 4.7 (1.7, 14.4) | 1.8 (0.8, 3.9) | 0.473 | 3.3 (1.6, 7.0) | 0.9 (0.3, 2.5) | 0.041* |
| Age group  (>85 years vs younger) | 3.1 (1.2, 8.7) | 1.6 (0.8, 3.5) | 0.182 | 2.7 (1.3, 6.0) | 1.8 (0.6, 5.3) | 0.736 |

* p for interaction, representing the heterogeneity of each estimate between CIRS groups
